# Evaluation of acupuncture treatments of postpartum female pelvic floor dysfunction by four-dimensional transperineal pelvic floor ultrasound

**DOI:** 10.1097/MD.0000000000027236

**Published:** 2021-10-22

**Authors:** Liping Yao, Fengzhi Li, Dandan Wang, Shaoqin Sheng

**Affiliations:** The Second Affiliated Hospital of Zhejiang Chinese Medical University, Hangzhou, Zhejiang, China.

**Keywords:** acupuncture therapy, evaluation, four-dimensional pelvic floor ultrasound, pelvic floor dysfunction, postpartum

## Abstract

**Introduction:**

In the present investigation, a systematic evaluation of the clinical treatment performance of diagnosed with pelvic floor dysfunction is explored. By comparing the 4Dtransperineal pelvic floor ultrasound images with the acupuncture treatment performance of the patients, an evaluation system with various parameters is established to provide critical information to guide the clinical treatment fpostpartum female pelvic floor dysfunction (FPFD).

**Methods:**

Eighty patients diagnosed with FPFD are divided into 2 groups. After the designated treatment to the patients, they are carefully examined using transperineal pelvic floor ultrasound. The shape and activity of bladder neck, cervix and rectum anal canal under resting, anal sphincter and Valsalva movements are observed and recorded. The morphology and continuous shape of levator ani muscle in different states after 4D image reconstruction are obtained.

**Results:**

After the acupuncture treatment, the bladder neck descent is decreased by 3.8 cm and the anal levator muscle area is decreased by 3.4 cm^2^ comparing with the control group. The anal levator muscle hole diameter is decreased by 0.3 cm, while the anterior and posterior diameter is reduced by 0.5 cm. Reduced possibility of cystocele and uterine prolapse is demonstrated by X^2^ test. These changes upon acupuncture therapy are in line with the improved conditions of the patients, indicating these parameters can help evaluate the therapy performance.

**Conclusion:**

4D pelvic floor ultrasound imaging provides objective and quantified information for the clinical diagnosis and treatment of FPFD and the assessment of therapy efficacy, making it a promising novel method in practical applications.

## 1 Introduction

Sharply increased incidence rate of female pelvic floor dysfunction (FPFD) has been observed in mainland China in recent years after the deregulation of One-child Policy after its implementation for decades. This change has attracted much attention to developing a novel treatment of FPFD that could be commonly applied at a low cost. It is the weak support of women's pelvic floor support tissues due to degeneration, trauma and other factors, resulting in pelvic floor dysfunction, which mainly include stress urinary incontinence, pelvic organ prolapse and female sexual dysfunction. The common pelvic floor supporting tissue is the muscles and ligaments of the pelvic floor. The general causes include a history of prolific birth, a history of dystocia, prolonged labor, or insufficient rest after delivery, or engaging in heavy physical activity, which leads to prolapse of the pelvic organs. Commonly practiced clinical examination methods for diagnosis include POP-Q classification, cotton swab test and urinary power test. At current stage, it is actually challenging to achieve an accurate clinical diagnosis of the patients and efficacy evaluation. One of the reasons is that the diagnosis and the evaluation mainly rely on patients’ clinical symptoms, physical examination and other physiological index. The accuracy of these approaches are often subjective and limited by the interpretation of the obtained results. More accurate and thus more reliable results may be given by magnetic resonance imaging study, but its application is impeded by the high examination cost,^[1,2]^ making it less applicable for post-natal examinations. Therefore, it is desired to directly and comprehensively probe and understand the structure and function of pelvic floor.

In recent years, the understanding of pelvic floor imaging studies has been improved thanks to the investigations and optimizations by lots of researchers.^[3]^ Novel ultrasound technology has broad application prospects in pelvic floor morphology examination, especially the 3D multi-section and 4D dynamic image acquisition techniques. Thanks to their powerful data post-processing capacity, pelvic floor anatomy could be clearly displayed and can thus be applied to pelvic floor dysfunction. The spatial resolution of the pelvic floor tissue and organs by the four-dimensional ultrasonography is close to that of magnetic resonance imaging.^[4]^ Its real-time feature makes it possible to further speed up the examination process and thus make it an even more desirable technique. The 4D view off-machine analysis can be exploited to reconstruct any three-dimensional plane image. The data collected from the pelvic tissue is measured in the corresponding physiological action state. The image playing back function enables the demonstration of the resting state as well as the movement pattern of the pelvic floor tissue and organ during the Valsalva movement. The comparison of the images collected at different periods helps the researchers to capture the image of the most serious state when the pelvic floor tissue is prolapsed. This method is simple and easy to popularize. In addition, four-dimensional ultrasound can reconstruct the plane of the levator ani muscle, visually showing the overall shape and continuity of the puborectal muscle. The information obtained at this conditions is critical for a full diagnosis since it is challenging to intuitively and accurately measure the extent of puborectal muscle tear, and check the anatomical structure of the pelvic floor by conventional clinical examination.

Traditional FPFD treatment methods often combine the application of biofeedback, pelvic floor muscle training, drugs and surgeries. Recently, the research on the treatment of female pelvic floor dysfunction by Chinese medicine as a complementary and alternative approach has attracted attention.^[5,6]^ For patients with pelvic floor dysfunction in China, acupuncture treatment as a traditional medicine has the advantages of smaller trauma and simpler operation, making it an alternative treatment option with low cost and high efficiency. Acupuncture is a general term for acupuncture and moxibustion. However, the theoretical study on the mechanism of acupuncture is rare, impeding its further development despite of the improved symposium of the patients in practical applications.^[7]^ For instance, acupuncture has shown a positive effect on relieving pain and postoperative nausea.^[8]^ Curative effects of acupuncture treatment on rheumatism, fatigue, neurasthenia, rectal prolapse, uterine prolapse have also been observed, which are especially significant for female pelvic floor dysfunction. Hence, we hypothesized that acupuncture can be used as a complementary or alternative route to improve FPFD in traditional physiotherapy, and four-dimensional transperineal pelvic floor ultrasound can be applied for the comprehensive diagnosis of FPFD after acupuncture treatment.

## 2 Subjects and methods

### 2.1 Patients and criteria

The patients participating in the present study were all diagnosed as pelvic floor dysfunction after 8 weeks of delivery from September 2018 to September 2019. The current investigation was reviewed and approved by The Second Affiliated Hospital of Zhejiang Chinese Medical University review board, and the registration number is ZYQT-20180349. All participants were consent for the current study. Some criteria were applied when carrying out the research.

Sample size was calculated using StatBox-Online Statistical Computing System: significance level 0.05, power of the study 80% and ratio of case to control equal to 1:1. It is hypothesized that there will be an 80% reduction in undesired cases in patients after intervention with acupuncture treatment compared to 50% in the control group. The sample size was estimated to be 36 patients and 36 controls. To compensate for patients withdrawing consent, 80 patients were planned for inclusion.

The patients must meet the following criteria:

1. age ≤40;
2. 8 weeks after delivery;
3. without symptom of pelvic organ prolapse, urinary fistula, history of pregnancy abortion, neuromuscular disease or history of pelvic surgery in the past;
4. normal weight of the newborn;
5. diagnosed as FPFD by a pelvic gynecologist based on the patient's clinical performance, physical examination and some special examinations (pad test, acupressure test, etc.);
6. the acupuncture treatments of all the cases were accomplished by the same personnel;
7. acknowledged the purpose and the procedures of this research and signed research informed consent.

Some exclusion criteria were also included in the present work. The candidate would be removed from this research if she has

1. vaginal bleeding or postpartum lochia persisted;
2. cannot be treated with acupuncture;
3. had pelvic floor rehabilitation exercise or pelvic surgery;
4. urinary incontinence caused by nervous system;
5. unable to complete effective pelvic floor muscle contraction or Valsalva maneuver.

### 2.2 Randomization, blinding, and treatment methods

All FPFD patients were randomly assigned to the treatment group (40 examples) or the control group (40 examples) by a computer-generated list using SPSS 20.0 software. Allocation was not revealed until each participant had completed their baseline assessment, at which time the participant were informed of their group assignment. Conventional physiotherapy of all the cases was accomplished by the same physical therapist blinded to the group assignment. Acupuncturist was the only study members aware of participant's group allocation.

All the patients in both the treatment group and the control group were given conventional physiotherapy. The first is to exercise their coordination ability. The supine position was taken on the bed, the knees were flexed and deployed outwards, and the palms were placed flat on the bed. The patients conducted 5 quick perineal contract movements, then rested for 10 seconds. These actions were repeated another 4 times. The exercise on strength and endurance were also executed at the same position. A contraction of the perineal muscles was maintained for 10 second while maintaining natural breathing followed by a break of ten seconds. These actions were repeated another 9 times. The above exercises were repeated for 3 times per day for 4 weeks.

Those in the treatment group were given extra acupuncture treatment using disposable acupuncture needles every day during the treatment period. Needles were retained at 5 acupoints in Zhongwan, Tianshu, Guanyuan, Qihai, and Zusanli, 2 acupoints for Pangguangshu and Shenshu were added with moxibustion, and 2 electroacupoints for Zhongji and Shuidao. The length was 40 mm and the diameter was 0.3 mm. After routine disinfection, the needle was straightly inserted at the perineal point by3 cm and rotated for 2 to 3 minutes. It was kept in the body for 30 minutes before removed. This treatment is practiced on a daily basis.

The statistical analysis of the data in this work is carried out by using SPSS 20.0 statistical software. The tests were conducted at a confidence interval of 95% (α = 0.05). The measurement data were expressed as Mean ± SD. Independent sample *t*-test was used in comparisons between 2 groups. *P* < .05 was considered statistically significant.

### 2.3 Ultrasonography techniques and diagnostic criteria

The ultrasonic images were collected using a GE Voluson E8 Color Doppler Ultrasound Machine equipped with RIC 5–9-D and RAB 6-D Probes. The working frequency was 5 to 10 MHz.

Prior to the image collection, urine and feces of the patients were removed. The amount of residual urine in the bladder must be <50 mL. The supine bladder lithotomy position was chosen while flexing the hip and gently abducting the 2 hips. The volume probe was outsourced to a condom, which was coated with disinfectant and chelating agent on both the inside and outside surfaces. The probe was firmly placed in the perineum of the patient. Two-dimensional and four-dimensional images were collected in both the resting and maximum Valsalva action (forced breath) conditions. The stored images were reconstructed and viewed using the 4D View analysis software.

**Figure 1:**
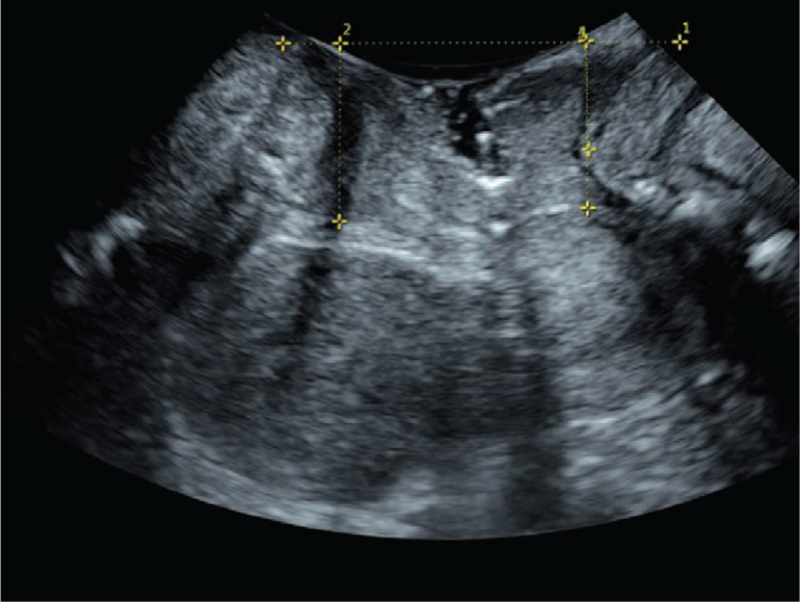
Ultrasonic image taken in resting state. ^∗^ Represents that there is a significant difference in the decrease of group B compared with group A (*P* < .05). Number 1 represents draw a horizontal line through the lower edge of the pubic symphysis in a resting state. Number 2 to 4 represent the distance measurements from the lowest point of the bladder neck (number 2), the lowest edge of the cervix (number 3), and the lowest point of the rectal ampulla (number 4).

**Figure 2:**
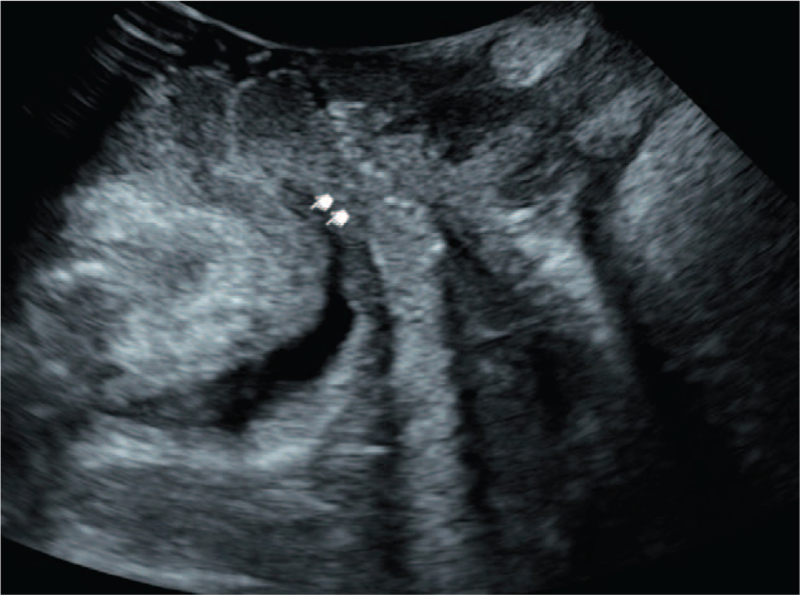
Ultrasonic image taken in the state of Valsalva maneuver.

**Figure 3:**
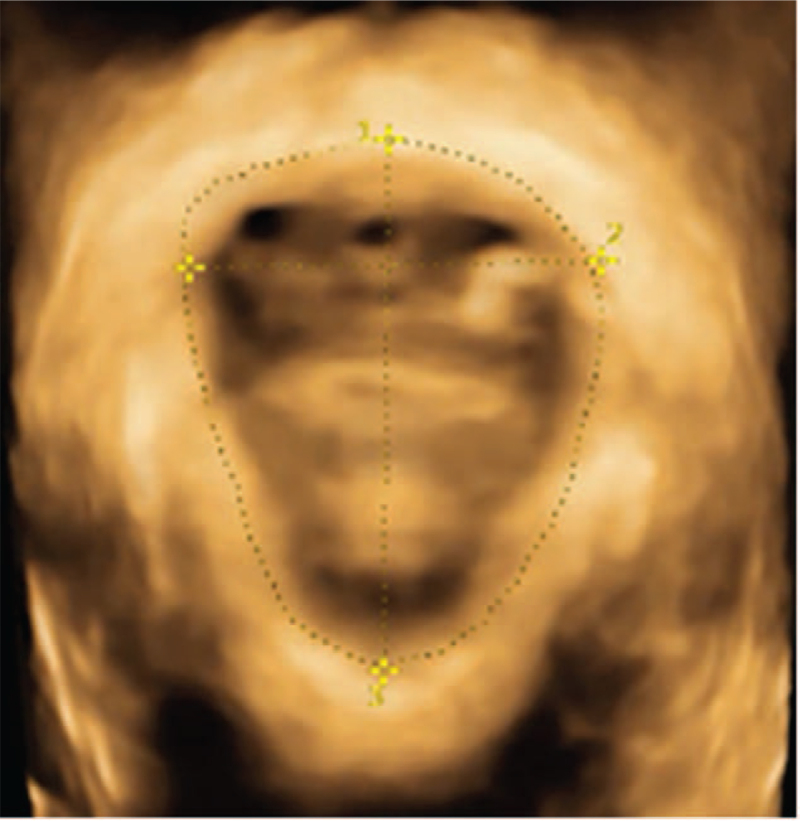
Ultrasonic image taken in resting state. ^∗^ Represents that there is a significant difference in the decrease of group B compared with group A (*P* < .05). Schematic diagram of the measurement of the area for the levator ani hiatus (number 1), left and right diameters (number 2), and anteroposterior diameters (number 3) in the resting state (all measured values are normal).

**Figure 4:**
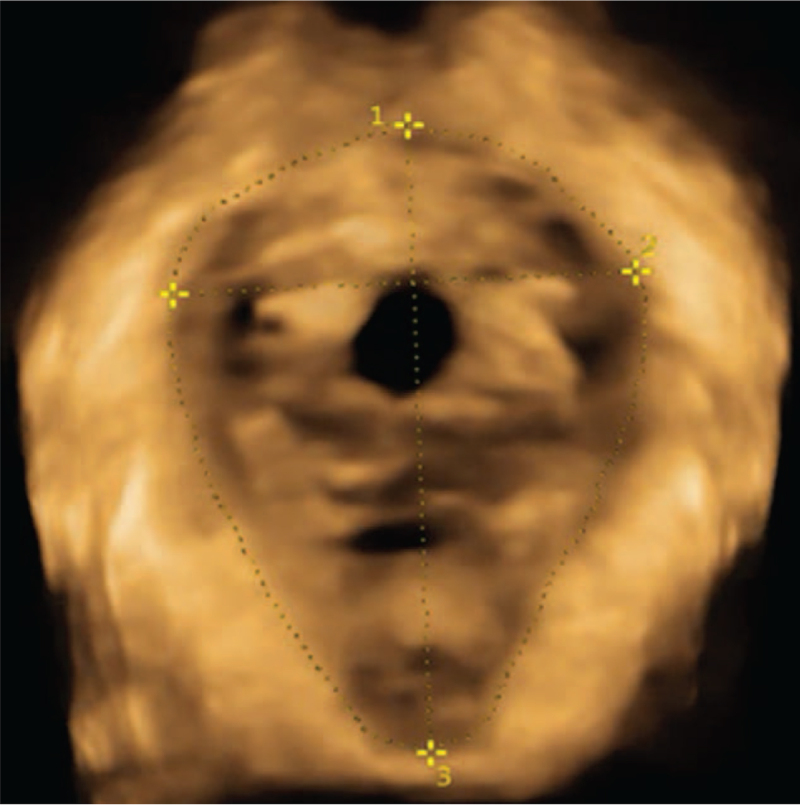
Ultrasonic image taken in the state of Valsalva maneuver. ^∗^ Represents that there is a significant difference in the decrease of group B compared with group A (*P* < .05). Schematic diagram of measurement for levator ani hiatus area (number 1), left and right diameter (number 2), and anteroposterior diameter (number 3) under Valsalva action state (each measured value increases).

Correct pelvic floor muscle contraction and Valsalus maneuver guidance training before ultrasound examination to ensure the accuracy of measurement parameters. The pelvic floor organisation index measurement was executed using the ultrasonic instrument. First, two-dimensional ultrasound on the median sagittal section of the pelvic floor shows the lowest point of the bladder neck at rest, the lower edge of the cervix, and the position of the rectum and abdomen. Second, after the pubic symphysis, the lower edge of the pubic symphysis was used as a horizontal line to measure the distance between the lowest point of the bladder neck, the lowest edge of the cervix and the lowest point of the rectum of the rectum in resting and Valsalian conditions (see Figs. 1–2). The relative mobility values of the bladder neck, cervix and rectum ampulla were calculated. After that, the four-dimensional ultrasound images were analyzed to calculate the area of the levator ani muscle rupture and the area of the levator ani muscle under the Valsalva motion. The diameter of the anal levator muscle hole as well as the anterior and posterior diameter were also measured (Figs. 3–4). Finally, the thickness and continuity of bilateral puborectalis muscles in the anal sphincter state were observed and recorded. The morphology and continuity of the levator ani muscle and the internal and external sphincter muscles were also observed and recorded.

## 3 Results

### 3.1 The conditions of the patients

Before evaluating the effect by ultrasonic treatment, it is necessary to rule out the effects due to the different conditions of the patients themselves. By comparing the general information of the patients participating in this research, the patients in the treatment group and the control group have no significant difference in terms of in age, current weight, gestational weeks, pregnancy weeks and neonatal weight between the 2 groups at a significance level of 0.05. The details are tabulated in Table 1.

**Table 1:** General conditions of the patients.

Parameters	Treatment group	Control group
Age	27.0 ± 5.3	27.2 ± 5.0
Current weight/kg	55.2 ± 4.3	54.7 ± 5.7
Gestational weeks/wk	39.1 ± 1.1	39.0 ± 1.2
Pregnancy weight/kg	68.0 ± 3.0	67.7 ± 5.7
Neonatal weight/kg	3.5 ± 0.5	3.4 ± 0.5

### 3.2 Effect of acupuncture treatment

After the acupuncture treatment, the number of undesired cases among the patients is reduced. In the control group where the patients only take regular exercises, 13 out of 40 have suffered systocele, while only 5 in the treatment group have this problem after receiving the acupuncture treatment. A similar phenomenon is observed when comparing the cases of uterine prolapse. The number of patient with uterine prolapse is decreased from 15 to 7 upon acupuncture treatment. By calculating the X^2^ of these parameters and the corresponding *P* values, it is revealed that there are significant differences between 2 groupsat a significance level of 0.05. It is thus proposed that the acupuncture treatment has a positive effect on relieving the postpartum female felvicfloor dysfunction. This observation encourages us to carry out further investigations to obtain more insights of the contribution of acupuncture treatment and achieve a more comprehensive understanding of the mechanism. Therefore, four-dimensional transperineal pelvic floor ultrasound is employed in this study for the critical information taking place upon acupuncture treatment.

### 3.3 Investigation by ultrasonic study

Four-dimensional transperineal pelvic floor ultrasound images are taken to compare the pelvic floor related parameters of the 2 groups, which displayed a significant difference in the postpartum female felvicfloor dysfunction symptom. The procedures are described in Section 2 and some representative graphs are shown here. Figure 1 shows the ultrasonic image taken in the resting state. The lower edge of the pubic symphysis was made into a horizontal line, and the distance from the lowest point of the bladder neck, the lowest edge of the cervix and the lowest point of the rectum of the rectum was measured. Figure 2, on the other hand, demonstrates the image taken under the action state of Valsalva. The inner neck of the bladder opens, forming a funnel shape, allowing urine to flow out from the inner mouth. As a result, a clinical urinary incontinence is observed.

Figure 3 is a schematic diagram of the measurement of the measurement of the area of the levator ani muscle in the resting state, the left and right diameter of the levator ani muscle, and the anteroposterior diameter of the levator ani muscle. Figure 4 is aschematic diagram of these measurement in Valsalva's action state. All measurements are normal in this image. The patients’ puborectalis muscles in the 2 groups on both sides result in normal thickness and complete continuity. Their levatorhiatus, internal and external anal sphincter result in normal shape and complete continuity.

When the ultrasonic images are taken, the data is processed following the aforementioned procedures to obtain the pelvic floor related parameters, which provide insights of the effect of acupuncture treatment. Their bladder neck descent (BND), anal levator muscle area, anal levator muscle hole diameter and anterior and posterior diameter values are paid special attention. They obtained parameters including the mean values and the standard deviations are displayed in Table 3. The *t* values are also calculated to determine whether a difference exists between the 2 groups. To support the hypothesis that there is no significant difference between the 2 groups with 40 measurements in each group, the t value must be smaller than 2.086. Apparently, the *t* values in Table 2 are all beyond this critical value. This phenomenon indicates the presence of significant differences in terms of BND, anal levator muscle area, anal levator muscle hole diameter and anterior and posterior diameter upon acupuncture at a significance level of 0.05.

**Table 2:** Effect of acupuncture treatment.

	Cystocele	Uterine prolapse	Rectocele
Control group	13	15	1
Treatment group	5	7	0
X^2^	4.588	4.013	–
*P*	.032	.045	–

**Table 3:** Comparison pelvic floor related parameters.

	BND/cm	Anal levator muscle area/cm^2^	Anal levator muscle hole diameter/cm	Anterior and posterior diameter/cm
Treatment group	16.4 ± 3.8	19.7 ± 1.9	4.5 ± 0.4	5.9 ± 0.3
Control group	20.2 ± 4.8	23.1 ± 2.6	4.9 ± 0.2	6.4 ± 0.5
*t* value	5.531	7.19	5.389	5.857

## 4 Discussions

FPFD is caused by congenital pelvic floor tissue and tissue degradation. It may also be triggered by acquired pelvic floor tissue damage due to acquired anatomical changes of pelvic organs. FPFD is manifested by a variety of symptoms, such as stress urinaryincontinence, overactive bladder, fecal incontinence, pelvic organ prolapse, sexual dysfunction, chronic pelvic pain. Among them pelvic organ prolapse and stress urinaryincontinence are the most common. FPFD can gradually occur and aggravate, affecting the quality of life of patients. The diagnosis and treatment of pelvic floor dysfunction often begins after the patient has obvious clinical symptoms. Most of the patients are already in perimenopausal period. As a consequence, the symptoms of dysuria and pelvic organ prolapse have been maintained for many years before the treatment. Diagnostic and non-surgical interventions, for instance, pelvic floor function training, can effectively delay the occurrence of severe pelvic floor dysfunction and improve the quality of life of patients. Therefore, early detection of FPFD has very important clinical significance. In developed countries, the proportion of patients attending female pelvic organ prolapse (female pelvic organ prolapse, one type of FPFD) each year is 38% to 76%, of which 10% to 20% require surgery.^[9,10]^ At present, the etiology of female pelvic organ prolapse is not very clear and may be related to many factors, such as genetic factors, obesity, and asthma. But pregnancy and childbirth are 2 important risk factors for pelvic floor dysfunction.^[11]^ Some studies have found that the pelvic floor damage caused by production is closely related to the prolapse of the anterior chamber and the middle chamber,^[12,13]^ while it is less correlated to anal prolapse.^[14,15]^ With the continuous improvement of women's social status and self-awareness all over the world, FPFD has become a social problem that cannot be ignored due to its high incidence. These symptoms have a wide-ranging impact on the quality of life of women, ranging from mild discomfort to paralysis and even severe functional impairment.^[16]^ Pelvic floor ultrasound can perform imaging evaluation of female pelvic floor function to prevent and reduce the occurrence of early FPFD. At the same time, it can evaluate the pelvic floor rehabilitation effect, guide clinical early pelvic floor muscle training to improve the pelvic floor muscle strength. The early diagnosis and early treatment guided by four-dimensional transperinealpelvic floor ultrasound study would benefit controlling the disease at the early stage, which could become a new strategy in dealing with FPFD.^[17]^ The feasibility for the observation of pelvic floor muscles and levator ani muscle through three-dimensional ultrasound method has been reported in literature.^[18]^ Weinstein et al^[4]^ found that perineal three-dimensional ultrasound is a reliable and accurate means to evaluate and measure levator ani muscle and puborectalis muscle. Braekeen et al^[19]^ used the perineal three-dimensional ultrasound to measure the area of the levator ani muscle, anteroposterior diameter, diameter and length of the levator ani muscle with good repeatability and accuracy. Based on the previous studies, the positions of various organs in the pelvic cavity often recover to the pre-pregnancy state about 6 weeks after delivery, which is convenient for assessing the short-term effects of childbirth on pelvic floor function. Perineal three-dimensional ultrasound was also applied to assess the extent of pelvic prolapse.^[20]^ It was also used to observe the morphology and function of the pelvic floor muscles and the changes in the pelvic floor structure of women with pelvic floor dysfunction.^[21–23]^ Despite of these studies on the structure and function of pelvic floor, the investigation on correlating the pelvic floor parameters with FPFD upon treatment is sparse. Particularly, the effect of acupuncture treatment on the structure of pelvic floor has not been explored through ultrasonic imaging to the best knowledge of the authors. The present work is thus focused on getting a better understanding this effect by four-dimensional transperinealpelvic floor ultrasound.

It has been noticed that increased BND is closely associated with female stress urinary incontinence.^[24,25]^ Among women with pelvic floor dysfunction, increased bladder neck mobility and vaginal delivery are also associated.^[26]^ In this study, the BND of the control group (20.2 ± 4.8) was significantly higher than that of the acupuncture treatment group with a BND of 16.4 ± 3.8. The perineal 4D ultrasound could visually show an increment in bladder neck mobility and measure its moving distance. Ultrasound examination has revealed 18 cases of bladder bulging, where 13 cases are in the control group and 5 cases are in the acupuncture group. All of them have BND values above 25 cm. Different degrees of stress urinary incontinence are also observed in these patients. This observation indicates that increased bladder neck mobility detected bypostpartum pelvic floor ultrasound examination is capable of providing objective and accurate data support for urinary abnormalities.

In this study, the maximum area of the levator ani muscle, the left and right anterior diameter of the levator ani muscle, and the anteroposterior diameter in the state of Valsalvamaneuver are clearly different after the acupuncture treatment. The area of the levator ani muscle of the acupuncture group is 19.7 cm^2^, which is much smaller than the 23.1 cm^2^ in the control group. Besides the area of the levator ani muscle, the ovarian levator muscle hole diameter and the the anteroposterior diameter of the levator ani muscle are also reduced from 4.9 and 6.4 cm^2^ to 4.5 and 5.9 cm^2^, respectively. Taking the improved synposym upon acupuncture treatment into consideration, the changes of these parameters may be related to the treatment performance. Dietz et al^[27]^ has reported the correlation between the levator ani muscle area in relation to the occurrence of pelvic organ prolapse. Dietz et al^[18]^also showed an increased likelihood of pelvic organ prolapse when Valsalva had an levator ani muscle area greater than 25 cm^2^. These studies illustrate the facility of evaluating the situation of FPFD with the pelvic parameters by ultrasonic imaging method, which is carried out in the present work. It is observed that the maternal pelvic floor abnormalities such as BND increase, bladder bulging, uterine prolapse and rectal bulging occurred 8 weeks after birth are suppressed by the acupuncture treatment. At the same time, the maximum area, left and right diameter and anteroposterior diameter of the Valsalva levator ani muscle were significantly smaller upon acupuncture treatment comparing with the control group. These observations illustrate that that the maximum area of the levator sac in the Valsalva movement may be closely related to the occurrence of postpartum pelvic dysfunction. Therefore, the perineal levator hole area, left and right diameter and anteroposterior diameter in the state of Valsalvamaneuver obtained by four-dimensional ultrasonography are criticalto guide the maternal pelvic floor dysfunction. Routine physical exercise therapy combined with acupuncture treatment could strengthen the pelvic floor muscle strength, accelerate the recovery of the relaxed pelvic floor muscles, and achieve the therapeutic goal of pelvic floor function rehabilitation of the pelvic floor dysfunction maternal. The results of this study show that the clinical efficacy of acupuncture combined with pelvic floor muscle training for postpartum women with pelvic floor dysfunction is more effective than sole pelvic floor muscle exercise.

## 5 Conclusion

In summary, the four-dimensional pelvic floor ultrasound can accurately identify the anatomical structure of the pelvic floor and dynamically observe the changes of the pelvic floor structure, providing an objective basis for early diagnosis of pelvic floor dysfunction and prevention, intervention and evaluation of therapeutic effects. Four-dimensional pelvic floor ultrasound is utilized to observe the changes of female pelvic floor structure after acupuncture treatment. The imaging indicators that comprehensively and accurately evaluate the efficacy of pelvic floor acupuncture are explored to provide a realistic and reliable imaging basis for the clinical practice. By combining acupuncture treatment with conventional physical exercise therapy, the pelvic floor anatomy of patients with postpartum pelvic dysfunction is effectively improved, which is critical to improve the patients’ quality of life. Moreover, the advantages of acupuncture treatment such as small trauma and easy operation make it worthy of further promotion for more practical applications.

## Author contributions

**Conceptualization:** Liping Yao.

**Data curation:** Fengzhi Li.

**Investigation:** Liping Yao, Dandan Wang.

**Methodology:** Liping Yao, Dandan Wang.

**Resources:** Shaoqin Sheng.

**Software:** Dandan Wang.

**Supervision:** Fengzhi Li.

**Validation:** Fengzhi Li.

**Visualization:** Fengzhi Li.

**Writing – original draft:** Liping Yao.

**Writing – review & editing:** Liping Yao.
